# Pediatric Plastic Bronchitis: Case Report and Retrospective Comparative Analysis of Epidemiology and Pathology

**DOI:** 10.1155/2013/649365

**Published:** 2013-04-11

**Authors:** Rebecca Kunder, Christian Kunder, Heather Y. Sun, Gerald Berry, Anna Messner, Jennifer Frankovich, Stephen Roth, John Mark

**Affiliations:** ^1^Departments of Pediatrics, Stanford University School of Medicine, Palo Alto, CA, USA; ^2^Departments of Pathology, Stanford University School of Medicine, Palo Alto, CA, USA; ^3^Departments of Otolaryngology, Head & Neck Surgery, Stanford University School of Medicine, Palo Alto, CA, USA

## Abstract

Plastic bronchitis (PB) is a pathologic condition in which airway casts develop in the tracheobronchial tree causing airway obstruction. There is no standard treatment strategy for this uncommon condition. We report an index patient treated using an emerging multimodal strategy of directly instilled and inhaled tissue plasminogen activator (t-PA) as well as 13 other cases of PB at our institution between 2000 and 2012. The majority of cases (*n* = 8) occurred in patients with congenital heart disease. Clinical presentations, treatments used, histopathology of the casts, and patient outcomes are reviewed. Further discussion is focused on the epidemiology of plastic bronchitis and a systematic approach to the histologic classification of casts. Comorbid conditions identified in this study included congenital heart disease (8), pneumonia (3), and asthma (2). Our institutional prevalence rate was 6.8 per 100,000 patients, and our case fatality rate was 7%.

## 1. Introduction: Index Case

Plastic bronchitis, an uncommon condition of obstructive airway casts, has been reported in adults and children, predominantly in association with an underlying cardiac or pulmonary pathology. In order to illustrate the disease course and the array of therapeutic options, we present an index case. This case, which also illustrates a novel multimodal approach to plastic bronchitis treatment, is one of the 14 identified through a search of the electronic medical record at our institution over a 12-year period. We report a systematic comparison of cast histology as well as calculation of plastic bronchitis prevalence and mortality.

The index patient, a 3-year-old male at the time of diagnosis with plastic bronchitis, was diagnosed with hypoplastic left heart syndrome by fetal echocardiography. Within hours of birth, he developed severe hypoxemia and underwent subsequent cardiac procedures including modified Norwood with placement of a right ventricle-to-pulmonary artery conduit, modified Blalock-Taussig shunt, bidirectional Glenn shunt, and extracardiac nonfenestrated Fontan.

**Figure 1:**
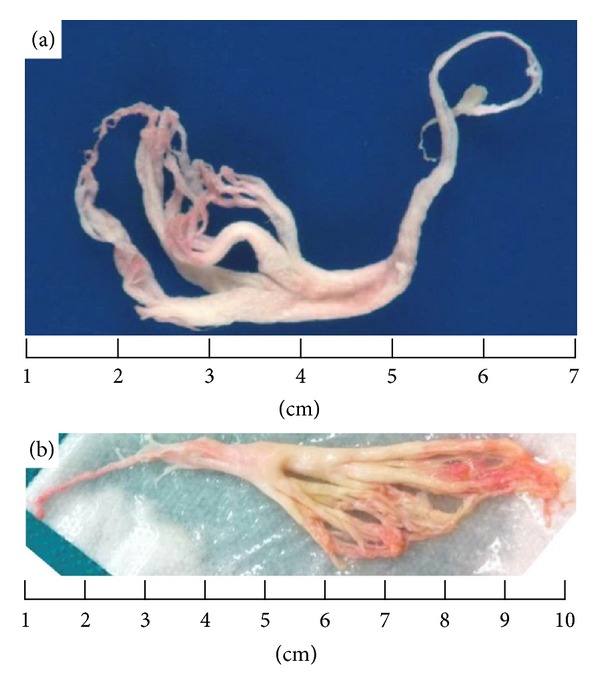
(a) Expectorated cast. (b) Extracted cast from rigid bronchoscopy.

**Figure 2:**
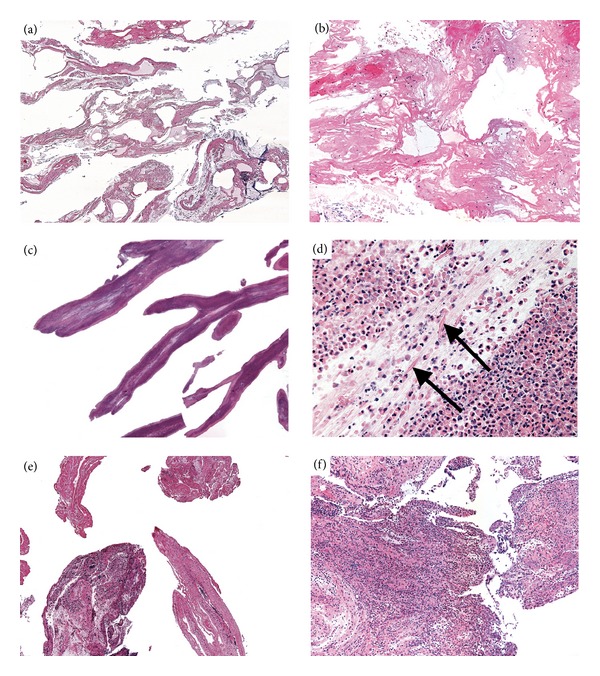
Histology from representative casts. Hypocellular fibrinous casts from patient 1 (case vignette) at 10x magnification (a) and 100x (b). Inflammatory casts from patient 9 showing eosinophils, Charcot-Leyden crystals and mucin at 10x (c) and 200x (d) arrowheads indicate Charcot-Leyden crystals. Casts from patient 3, showing hypocellular ((e), 40x) and inflammatory ((f), 100x) sections in the same specimen.

 Pre-Fontan cardiac catheterization at 27 months of age showed normal pulmonary vascular resistance and an unobstructed Glenn circuit. After Fontan surgery, the patient developed hypoxia and low cardiac output from presumed intraoperative right lung injury. He required venoarterial extracorporeal membrane oxygenation (ECMO) for 5 days. The patient initially improved off of ECMO with normal Fontan pressures, but in the subsequent weeks his respiratory status declined. He developed increasing cough and 3 weeks postoperatively, expectorated a large, flesh-colored cast. The cast was spongy and consisted of irregular branches resembling the bronchial tree. Microscopic examination revealed fibrinous, hypocellular material (Figures 1 and 2). 

The patient continued to expectorate smaller casts on over the next month. His respiratory treatments included high-frequency chest wall oscillation (vest) chest physiotherapy, inhaled hypertonic saline, inhaled levalbuterol, and inhaled tissue plasminogen activator (t-PA). One month after the initial cast expectoration, he had a respiratory deterioration and was taken for rigid bronchoscopy. Despite bronchoscopic cast removal (Figure 1(b)), the patient had persistent right upper lobe atelectasis on chest radiograph as well as a worsening oxygen requirement. Therefore, he underwent repeat bronchoscopy (both rigid and flexible) 3 days after the first. During the second bronchoscopy, t-PA was directly instilled into the right upper lobe bronchus (15 mL of 1 mg/mL t-PA). A third bronchoscopy was performed 1 week later with cast removal as well as repeated direct t-PA instillation (5 mL of 5 mg/mL t-PA by suction catheter). No complications were associated with the t-PA administered during the bronchoscopies.

After these 3 bronchoscopies, the patient was weaned off supplemental oxygen and had improved aeration on exam and chest radiograph. In addition to the previously mentioned treatments, the patient was started on azithromycin (for anti-inflammatory effects) and spironolactone, which has been associated with improvement in protein-losing enteropathy after Fontan surgery [1]. High-resolution computed tomography (CT) with angiography revealed a patent Fontan pathway and no evidence of remaining casts or lung fibrosis. The patient was discharged home on inhaled t-PA (5 mg, 4 times daily), inhaled levalbuterol, oral azithromycin, inhaled budesonide, and vest treatments. At 6 months on these therapies, he continued to expectorate casts, but had significantly fewer episodes of airway obstruction. 

## 2. Methods

 The Stanford University Institutional Review Board approved this study. To identify patients with plastic bronchitis at our institution, we used a unique approach in which data were captured in our institution's electronic medical record (EMR) and a research data warehouse. The data storage platform, termed Stanford Translational Research Integrated Database Environment (STRIDE), acquires and stores all patient data contained in the EMR at our hospital and provides immediate semantic and navigational search capabilities. We identified 205,100 pediatric patients (birth to age 18 years) who were admitted to our hospital or seen in our clinics between January 1, 2000 and January 1, 2012. Using the STRIDE tool known as the “Anonymous Patient Cohort Tool,” we created a plastic bronchitis cohort through a semantic search of all dictated records. This same tool was also used to identify the following patient cohorts based on ICD (International Classification of Diseases) 9 codes: congenital heart disease (745, 746, 747), asthma (493), and pneumonia and/or influenza (480–488.9).

## 3. Results

Our electronic search revealed 13 patients with plastic bronchitis and 1 patient who was followed during the study time period but was diagnosed in 1999. All 14 cases of plastic bronchitis were diagnosed by the gross appearance of airway casts (either expectorated or removed by bronchoscopy). Seven cases were evaluated further by pathologic examination, and the histological findings are described in Table 1. All 14 patients had diagnostic imaging with findings suggestive of bronchial casts, including chest radiographs showing atelectasis and lobar collapse. Comorbid conditions included congenital heart disease (8), pneumonia (3), asthma (2), acute lymphocytic leukemia (1), systemic lupus erythematosus (1), sand aspiration (1), and tracheal sling (1). 

 Regarding the epidemiology of plastic bronchitis, the 14 plastic bronchitis patients were seen over a 12 years among 205,100 total patients seen at our institution during the study time period. The overall institutional prevalence rate was 6.8 per 100,000 patients. The prevalence rates of plastic bronchitis among the various patient cohorts were as follows: congenital heart disease (113 per 100,000), pneumonia and/or influenza (58 per 100,000), and asthma (29 per 100,000). There was only one death (patient 14) among the 14 patients resulting in a fatality rate of 7%.

Histopathologic review was performed on 14 specimens from 7 patients in this series. Three patients had a history of complex cyanotic congenital heart disease (patients 1, 2, and 3 in Table 1). The casts from patient numbers 1 and 3 were hypocellular and composed primarily of eosinophilic, fibrinous material, with a scant amount of mucin at the edges (Figures 2(a) and 2(b)). Most cells in these casts were mononuclear cells (lymphocytes and entrapped alveolar macrophages), although some granulocytes were present as well. Casts in the 2 asthma cases (patients 9 and 10, Figures 2(c) and 2(d)) were primarily cellular, composed of sheets of eosinophils with associated Charcot-Leyden crystals, and surrounded by mucin. One patient (patient 3) had a history of both complex cyanotic congenital heart disease and asthma, and his casts had a mixture of the 2 morphologic appearances described previously, with some hypocellular casts made mostly of fibrinous material, and other more mucinous casts with florid infiltrates of eosinophils (Figures 2(e) and 2(f)). Casts from 2 cases (patients 1 and 10) showed occasional bacterial microcolonies without associated inflammation, suggesting colonization versus infection.

Treatment modalities varied between patients (Table 1), but the majority of patients had bronchoscopic cast removal (*n* = 11). During bronchoscopy, 2 cardiac patients had direct instillation of dornase alpha (recombinant DNase), and 1 had direct instillation of t-PA (patient 1 described in case vignette). Commonly used inhalation treatments included corticosteroids (*n* = 8), dornase alfa (*n* = 4), and t-PA (*n* = 4). The patient described was the only one in this case series to receive both inhaled and directly instilled t-PA. Oral azithromycin was administered to 4 patients for its immunomodulatory properties. Inhaled beta agonists were administered to both cardiac and asthma patients, and oral spironolactone was started after the diagnosis of plastic bronchitis in 2 of the 8 cardiac patients. Of note, the patients with asthma required fewer medications to reduce cast formation compared to the patients with cardiac disease.

## 4. Discussion

### 4.1. Epidemiology

Plastic bronchitis is an uncommon condition, but recent evidence suggests that it is underreported as well [2]. Our report represents one of the largest case series and includes institutional prevalence rates of plastic bronchitis with selected cardiac and pulmonary diagnoses. The prevalence seen at our institution may be higher than other pediatric centers, as our center is a referral center for tertiary and quaternary care. Of note, plastic bronchitis prevalence in Fontan patients has been estimated to be as high as 4–14% [2]. The patients in our cohort can be placed into 2 main categories: (1) those with congenital heart disease, and (2) those with primary pulmonary processes (Table 1). Historically, these are the 2 most common diagnostic groups associated with plastic bronchitis. Due to the small number of cases reported, a gender or age predilection was not demonstrated. This is consistent with a retrospective survey study of Fontan-associated plastic bronchitis which showed no reliable clinical or demographic predictors for plastic bronchitis [2]. 

### 4.2. Presentation and Diagnosis

Plastic bronchitis was first reported by Galen (AD 131–200), who described the expectoration of “arteries and veins.” While the alarming presentation of large, branching, expectorated casts is pathognomonic, many patients present with less specific symptoms such as dyspnea, cough, and fever. Severe hypoxia due to airway obstruction can occur either on presentation or in the course of the disease. On physical exam, wheezing or decreased breath sounds are commonly observed in symptomatic patients. The auscullatory “flag snapping” sign (also called bruit de drapeau) is attributable to a partially obstructing cast moving in a bronchus [3]. Radiographic findings are often nonspecific and include atelectasis or infiltrate(s). A recent report demonstrated that contrast-enhanced chest CT can be used both to aid in diagnosis and determine the location of casts for bronchoscopic extraction [4]. Although noninvasive imaging can assist in the diagnosis, a cast specimen for gross and microscopic examination is usually required to confirm the diagnosis. If there is no history of cast expectoration in a patient at risk, a high index of suspicion is appropriate due to the rapid decompensation and even fatal outcome due to acute airway obstruction. The use of bronchoscopy for both diagnosis and treatment is important. *In situ* casts vary in size and can extend throughout the entire tracheobronchial tree. Expectorated or extracted cast material is generally beige to white in color and rubbery in consistency. The parents of the patient in the case presented believed that the early casts he expectorated at home were bits of string cheese that he had previously ingested. 

### 4.3. Histology

Seear initially proposed a classification based on the morphological composition of the mucus plugs [5]. According to this classification, type I casts are composed of inflammatory cells (e.g., neutrophils and eosinophils) and fibrin; they are more commonly associated with primary pulmonary disease and bronchial inflammation. Type II casts are hypocellular and consist predominantly of mucin; they are more commonly associated with congenital heart disease. In contrast to the type II casts defined by Seear, the type II hypocellular casts in our series were primarily composed of amorphous eosinophilic, fibrinous material rather than mucin. Also, the type I cellular casts we reviewed were mainly composed of mucin rather than fibrin. These findings are based on examination of conventional hematoxylin and eosin-stained sections; all were reviewed simultaneously in this retrospective review. Periodic Acid-Schiff with diastase (PASd) staining performed on casts from a patient with congenital heart disease (patient 1) confirms this finding, staining scant mucin only at the periphery of the hypocellular cast. A PASd stain of the cast from the patient with congenital heart disease and asthma (patient 3) showed increased mucin in the areas with type I cellular inflammatory cast morphology. 

Although we have reviewed a limited number of casts, our case series indicates that a subset of cases may not fit precisely into the Seear classification system. In our case series, the type I inflammatory casts, seen mainly in patients with asthma, are composed of sheets of eosinophils in a mucinous matrix. The type II hypocellular casts, seen in cases of congenital heart disease, contain scattered acute inflammatory cells and macrophages and are otherwise composed of fibrinous material. This is consistent with a recent prospective study of casts from congenital heart disease patients by Heath et al. showing them to be primarily fibrin [6]. The association of type I inflammatory casts with mucin and type II hypocellular casts with fibrin is an important observation, as it relates to pathophysiology, potential response to treatment, and diagnosis. 

### 4.4. Pathophysiology

The mechanism of cast formation remains unclear both for the inflammatory casts in lung disease and the hypocellular casts associated with congenital heart disease. To more fully account for underlying disease and explore the pathogenesis of cast formation, Brogan examined the role of comorbid conditions: allergic/asthmatic, cardiac, and idiopathic [7]. In a series described by Madsen, the classifications were combined to integrate information from cast histology and patient history. Patients were divided first based on co-morbidity and then on cast histology if no underlying disease was identified [8]. This group reviewed all published cases of plastic bronchitis and noted that the purely histologic distinction was likely an oversimplification. Our data supports the Madsen classification system, but demonstrates that casts in congenital heart disease can be predominantly hypocellular and fibrinous.

Regarding the etiology of casts in patients with congenital heart disease, there is evidence that abnormal lymphatic drainage may have a role, as casts have been found both in primary lymphatic system disease and postcardiac surgery in association with protein-losing enteropathy and chronic recurrent chylothoraces [9, 10]. We speculate that the patient described in the case presented may have an underlying structural abnormality of the lymphatic vasculature in his lungs. This abnormality has been described at autopsy of infants with hypoplastic left heart syndrome and a highly restrictive or intact atrial septum resulting in high fetal pulmonary venous pressure [11]. Of the two patients in this series with hypoplastic left heart syndrome (patients 1 and 7), both had intact atrial septa.

Although the relative contribution of congenital lymphatic defects versus acquired or operative lymphangiectasia to cast formation is unclear, this case series supports the hypothesis of abnormal lymphatic drainage in patients with congenital heart disease contributing to the formation of casts. In particular, the casts from patients with congenital heart disease in this series are composed mostly of fibrinous material, which may be a result of plasma proteins contained in extravasated lymph. Said so, although these casts have the staining characteristics of fibrin and internal structure reminiscent of fibrin thrombi seen in blood vessels, routine histology is not specific for fibrin *per se*, as other proteinaceous materials could have a similar appearance. Proteomic analysis of casts would likely be informative in this regard.

It has also been suggested that elevated pulmonary venous pressure resulting in increased mucous production is responsible for cast formation [5]. However, Madsen notes that many of the cardiac conditions associated with plastic bronchitis do not result in elevated pulmonary venous pressure and that cast formation is not seen in other diseases with pulmonary hypertension [8]. In the patient in the case presented, for example, all available evidence suggested that his Fontan pathway pressures were within the usual range. Madsen et al. propose a 2-step model for cast formation wherein inflammation resulting in dysregulated mucus secretion is superimposed on a susceptible genetic background. Our patient may represent a different pathophysiology, given the lack of associated inflammation, and the minor contribution of mucin to the bulk of the casts.

In patients with asthma, the cause of casts is likely related to chronic inflammation and its attendant neutrophilic and eosinophilic airway infiltration. With decreased mucociliary clearance, the airways become occluded with eosinophils and neutrophils in a mucinous background [8]. The cases reported in this series support this hypothesis, given the predominance of inflammatory cells and mucin in casts from patients with asthma. Interestingly, the one case (patient 5) with both congenital heart disease and asthma had an intermediate cast composition with areas of hypocellular fibrin and other areas with inflammatory infiltrate.

### 4.5. Therapy

Both acute cast removal and long-term prevention of cast recurrence are the primary therapeutic objectives. Regarding mechanical cast disruption, flexible or rigid bronchoscopy is most often used for cast removal and can be guided by contrast-enhanced CT imaging. A large case series of 22 pediatric patients concluded that bronchoscopic extraction is the only effective modality for treatment [12]. A report of 2 patients for whom bronchoscopy was not an option showed that high-frequency jet ventilation can be used for short-term clearance of casts [13]. Chest physiotherapy is frequently employed as an adjunct for cast mobilization. 

A variety of inhaled mucolytics and fibrinolytics have been used for cast disruption. We report topical therapy during bronchoscopy using the fibrinolytic agent t-PA as was recently reported by Gibb et al. [14]. However, the majority of case reports involve inhaled t-PA for cast disruption [15–18]. Inhaled t-PA is used predominantly in cardiac patients, which is consistent with our finding that these casts have more significant fibrin content. The use of t-PA is supported by the results of Gansey, who incubated extracted casts from a Fontan patient with t-PA and observed complete dissolution of the cast [19]. It is further supported by Heath et al., who had similar observations with casts from 4 children with congenital heart disease [6]. Other fibrinolytics that have been aerosolized for use in plastic bronchitis include heparin and urokinase [19, 20]. Inhaled mucolytics including acetylcysteine and dornase alpha are commonly used in patients with plastic bronchitis [15, 21]. Dornase alpha has been applied topically during bronchoscopy and resulted in effective cast removal [22]. Our data suggest that mucolytics would be potentially most useful in type I inflammatory casts, which have a higher mucin content.

## Figures and Tables

**Table 1:** Clinical presentation, treatment, and outcomes of 14 patients with plastic bronchitis.

	Patient identification	Presentation of PB	Treatment	Gross description and histopathology	Outcome
1	3 yo M with hypoplastic left heart syndrome. Cardiac surgeries included Norwood, BT shunt, Glenn, and Fontan.	Worsening respiratory distress and expectoration of multiple casts after Fontan.	Multiple bronchoscopic cast removals, budesonide, levalbuterol, direct and inhaled t-PA, spironolactone, inhaled hypertonic saline (3%). Oral azithromycin and spironolactone.	Gross: irregular branching, spongy, soft, tan, and red-brown tissue. Largest specimen 6 × 1.5 × 0.7 cm. Histology: hypocellular fibrinous casts.	Continued small expectorated casts but without further obstructive casts 6 months after PB diagnosis.

2	3 yo M with tricuspid atresia. Cardiac surgeries included Glenn and Fontan. Course complicated by protein-losing enteropathy and chylothorax.	Presented with chronic cough; expectoration productive of branching mucoid casts.	Bronchoscopic cast removal. Inhaled steroids, albuterol, acetylcysteine, dornase alpha, and alteplase. Oral azithromycin.	Gross: 2.2 × 1.4 × 0.3 cm white fibrous tissue. Histology: hypocellular, fibrinous cast.	Continued levalbuterol and acetylcysteine with small expectorated casts daily 12 years after PB diagnosis.

3	6 yo M with d-transposition of the great arteries and asthma. Cardiac surgery included arterial switch, closure of ASD, VSD, and PDA ligation.	Respiratory distress and right lung collapse in setting of influenza B infection. Bronchoscopy followed by forceps removal of cast.	Bronchoscopic cast removal, inhaled budesonide, acetylcysteine, dornase alpha, levalbuterol, inhaled t-PA. Oral azithromycin.	Gross: thick, white, extremely viscous material adherent to bronchus wall and obstructing right mainstem bronchus. Histology: mixture of hypocellular fibrinous casts and inflammatory casts with abundant eosinophils.	Well-controlled asthma with no further casts at 3 years after PB diagnosis.

4	1 yo M with DiGeorge syndrome, tetralogy of Fallot, pulmonary atresia, and MAPCAs with chronic lung disease who was ventilator dependent. Cardiac surgeries included unifocalization to RV-to-PA conduit with VSD closure.	Repeated plugging of tracheostomy with thick mucous.	Inhaled dornase alpha, levalbuterol, albuterol, budesonide. Oral azithromycin.		Continued on inhaled dornase alpha, budesonide, levalbuterol, and albuterol at discharge, which was 2 months after initial diagnosis of PB.

5	2 yo M with tricuspid atresia. Cardiac surgeries included Glenn and Fontan. Course complicated by chylothorax.	Presented with significant cough which improved after expectoration of cast with delicate strands.	Budesonide, levalbuterol, spironolactone.		Fontan was fenestrated after PB diagnosis and no further casts at 9 months after PB diagnosis.

6	2 yo F with heterotaxy with atrioventricular septal defect and mitral regurgitation. Cardiac surgery included repair of septal defect and subsequent orthotopic heart transplant.	Acute inability to ventilate while intubated with left lung collapse 1 week after heart transplant.	Bronchoscopic cast removal by side-channel sucker.	Gross: thick, rope-like yellow mucoid secretions.	Resolved after cast evacuation with no further casts at 17 months after PB diagnosis.

7	3 yo M with hypoplastic left heart syndrome. Cardiac surgeries included Norwood with RV-to-PA conduit, aortic arch reconstruction, bidirectional Glenn, and extracardiac Fontan with subsequent Fontan takedown.	Persistent atelectasis and respiratory failure after Fontan takedown and return to Glenn physiology.	Bronchoscopic cast removal and direct instillation of dornase alpha. Inhaled albuterol, levalbuterol, and budesonide.		Discharged from hospital on albuterol, levalbuterol, and budesonide at 3 months after PB diagnosis.

8	4 yo M with tetralogy of Fallot, pulmonary atresia, and MAPCAs. Cardiac surgeries included right unifocalization to RV-to-PA conduit and left unifocalization to central shunt.	4 days after unifocalization revision left lung whiteout noted while patient was being mechanically ventilated.	Bronchoscopic cast removal using forceps. Direct and inhaled dornase alpha, inhaled t-PA.	Gross: tenacious mucoid material straddling the carina.	No further cast production after hospital discharge, which occurred 1 month after PB diagnosis.

9	2 yo M with moderate, persistent asthma complicated by pneumonia.	Presented with cough and wheeze with possible foreign body aspiration.	Bronchoscopic cast removal. Inhaled levalbuterol, montelukast, and budesonide.	Gross: several white to tan branching segments, largest 3.9 × 0.2 × 0.2 cm. Histology: mucinous casts with abundant eosinophils and scattered Charcot-Leyden crystals.	Occasional asthma exacerbation, but overall well controlled with no further episodes of cast formation 4 years after PB diagnosis.

10	15 yo M with exercise-induced asthma.	Presented initially to outside hospital with dyspnea and found to have left mainstem bronchus lesion of unclear etiology. Despite laser resection, obstruction recurred as did respiratory distress. Repeat bronchoscopy with diagnosis 4 months after initial presentation.	Bronchoscopic cast removal.	Gross: 3 × 2 × 0.3 cm irregular, slightly tubular tan, erythematous, soft material. Histology: mucinous casts with abundant eosinophils and Charcot-Leyden crystals.	Required further bronchoscopic cast removal, most recently documented 3 months after PB diagnosis.

11	9 yo M with history of mild asthma complicated by massive sand aspiration.	Respiratory arrest following massive sand aspiration. Bilateral pneumothoraces, bilateral lung collapse, and tracheal tear requiring ECMO support.	Bronchoscopic cast removal along with lung lavage.	Gross: dark, brown, irregular, hemorrhagic pieces of tissue, largest 3.2 × 1.5 × 0.5 cm. Histology: hypocellular, fibrinous casts with entrapped red blood cells.	Continued intermittent albuterol use, but no further casts 2 years after initial PB diagnosis.

12	17 mo F with tracheal sling. Tracheoplasty was complicated by prolonged intubation and tracheal stenosis.	After extubation, rigid bronchoscopy performed due to continued stridor which revealed early plastic bronchitis and tracheomalacia.	Inhaled tobramycin, acetylcysteine, levalbuterol, and budesonide.	Gross: thick, yellow, tenacious secretions.	Bronchoscopy 1 week after initial PB diagnosis without casts present.

13	19 yo F with ALL treated with matched sibling bone marrow transplant complicated by graft versus host disease. History of ASD and VSD after repair.	Acute respiratory distress during transplant hospitalization. Large obstructing mucous plug found on bronchoscopy.	Bronchoscopic cast removal using forceps.	Gross: 4 × 2 × 1.7 cm granular, tan-red-green fragment.	No further cast production with most recent followup 2 years after initial PB diagnosis.

14	19 yo F with systemic lupus erythematosus complicated by pulmonary hemorrhage, ARDS, CMV pneumonitis, and aspergillosis.	Initially intubated for pulmonary hemorrhage, but because required increasing pressures, flexible bronchoscopy was performed. This revealed cast in the right mainstem bronchus which was removed using rigid bronchoscopy.	Bronchoscopic cast removal.	Gross: 18 × 6.5 × 1 cm fibrinous cast. Histology: fibrinous casts with associated acute inflammation and hyphal forms.	Deceased 1 week after cast extraction due to persistent pulmonary hemorrhage resulting in cardiogenic shock.

ALL: acute lymphoblastic leukemia; ARDS: acute respiratory distress syndrome; ASD: atrial septal defect; BT: Blalock-Taussig; CMV: cytomegalovirus; ECMO: extracorporeal membrane oxygenation; F: female; M: male; MAPCAs: major aortopulmonary collateral arteries; PA: pulmonary artery; PDA: patent ductus arteriosus; PB: plastic bronchitis; RV: right ventricle; RVOT: right ventricular outflow tract; t-PA: tissue plasminogen activator; VSD: ventricular septal defect.
